# The Cancer Campaign

**Published:** 1915-07

**Authors:** James H. Lewis

**Affiliations:** Buffalo, N. Y.


### BUFFALO MEDICAL JOURNAL

Yearly Volume 70. JULY, 1915. No. 12

### ORIGINAL ARTICLES

The right is reserved to decline papers not dealing with practical medical and surgical subjects, and such as might offend or fail to interest readers. Contributors are solely responsible for opinions, methods of expression and revision of proof.

### The Cancer Campaign.

JAMES H. LEWIS, M. D., F. A. C. S., Buffalo, N. Y.

The cancer campaign has as its object the stimulation of the general practitioner to more activity in making early diagnosis of cancer and to encourage his insistence on early surgical treatment. Through the medical profession it is hoped to disseminate such knowledge of cancer among the laity that will bring them to the physician in the earlier stages of the disease. As has been said, the knowledge of the medical profession in one generation becomes the possession of the laity in the next; and if the profession itself comes to a due appreciation of the great importance of early recognition of this deadly scourge and the absolute necessity of early surgical removal in order to effect a cure, the laity will soon come to know the early signs of the disease and will present themselves when cure is possible. Illustrating the readiness with which the public takes up matters of this nature may be instanced the general appreciation now of the signs of appendicitis and the general willingness to submit to operation.

The total deaths from cancer in the registration area of the United States from 1901 to 1906 amounted to 140,088. In 1906 in the registration area which then represented a population of 31,292,130, there died 29,020 persons of cancer, or at the rate of 70.8 per 100,000 population. S. P. Beebe estimates that approximately 75,000 persons died in the United States during the past year from cancer. On the basis of English statistics for the year 1906 it was computed that 1 in 11 of all men and 1 in 8 of all women, 35 years of age and upward, eventually die of cancer. The majority of observers are of the opinion that cancer is on the increase. McGlinn has computed that with the increase of cancer and decrease of tuberculosis, in the year 1931 the cancer mortality will surpass that from tuberculosis.

Two facts have been established with reference to cancer, the importance of which cannot be too much emphasized, viz: (1) that the disease is at first and in the early stages local, and (2) that it soon spreads to other regions nearby and distant. On the first fact depends the curability of cancer by early surgical interference. To surgeons of a generation or more ago this was not clear. An almost continuous succession of failures to cure by operation blinded them to it. The cause of their failures they did not appreciate, namely, insufficient removal of tissue and operation at a time when metastases had already occurred. Sir James Paget is quoted as stating in his lectures on Surgical Pathology, "I will not say such a thing as cure is impossible, but it is so highly improbable that a hope of this occurring cannot be reasonably entertained." And this is now the view very generally held by the laity, and which it is our duty as far as lies in our power to eradicate in order that patients on their earliest realization of cancerous signs may present themselves for relief.

Another fact of importance bearing on the curability of cancer is that in about 80% of the cases it occurs at sites in the body where surgical intervention is possible. In woman the favorite sites are the breast and uterus, and in man the stomach, rectum, lip, mouth, tongue and skin are accountable for the majority of cases.

Do the results of present day surgery justify us in insisting on its efficacy as a means of treatment? Undoubtedly, yes. Contrast the practically unbroken succession of failures under all past modes of treatment with the substantial percentages of cures now being reported by operators generally. These cures for various sites range from 10 to 50%. And these percentages are obtained not merely in the most favorable cases, but in those considered "operable," that is, where the disease has not clearly gone beyond the possibility of complete removal. Statistics on breast cases have been worked out most fully and these show, almost uniformly, in operable cases, cures in over 20% of the cases and at the hands of many operators, over 30% of cures. As an example, take the series of 210 traced cases, reported by Halsted of Johns Hopkins, with the record of 75 cures or at the rate of 42.3%. Analyzing these cases Halsted reports that in 40 where there was glandular involvement in the neck and in the axilla there were 3 cures (7.5%), in 110 cases where there was glandular involvement in the axilla but not in the neck, there were 27 cures (24:5%), and (this should be particularly noted) in 60 cases where there was no glandular involvement in either neck or axilla, 45 cures or at the rate of 75%. The proof is clear that cancer can be eradicated by surgery and that the chances of success are by far the best in the early stages of the disease. Bloodgood, after a most careful study of 1300 cases of tumors of the breast concludes: "If every woman over 25 years of age were to seek surgical advice the moment she felt a lump in the breast and the surgeon explored this lump at once, the probabilities are that the lump would prove to be benign in about 30% of cases; in the malignant cases the chances are that the tumor would be adenocarcinoma in 20% of the cases with the probability of a cure of 100%. If the lump represented the most malignant forms of cancer, the chances are 85% of a cure." If cancers could be attacked at the first sign of their presence, the disease would still be local in the great majority of cases, and cure could be confidently expected. It is, however, but seldom that the surgeon sees the disease in its early stages, and too often the operable stage has passed. As stated by Wainwright: "Of 228 cases applying at the Johns Hopkins Hospital with breast cancer, 29% were inoperable on admission. Out of 100 cases of cancer of the uterus applying at the Vienna Clinics, 85% were inoperable." The cause of this delay is not that signs are absent, that the patient does not know that something is wrong. Illustrating this fact,—in two series of operable breast cases given by Geo. P. Childe, in his readable and forceful book "The Control of a Scourge," the average duration of the disease to the patient's knowledge, previous to coming into the hands of the surgeon, was well over a year. In 34 cases of cancer of the uterus, the patients had had to their knowledge signs of an average duration of 8 months previous to obtaining surgical aid. The pitiable fact is, that in the great majority of cases, the patients reach the surgeon after the disease has ceased to be local, when delay has rendered cure impossible. The disease has indicated its presence by definite signs, but through ignorance or carelessness on the part of patient or physician, the favorable time for cure has gone and there is nothing further but a period of suffering, and then death. As Childe says: "Cancer itself is not incurable. It becomes incurable from the simple fact that its unfortunate victims harbor and nurse their cancers till it is too late. It is not the disease which is incurable, it is the delay which makes it so." In the consideration of cancer we must rid our minds of the time honored picture of the disease in which the prominent features are pain, foul discharge, hemorrhage, loss of flesh and strength and cachexia. When this stage is reached the patient is doomed—all hope of cure is gone; operation is as useless except occasionally in palliation as it is to lock the stable door after the horse is stolen.

Cancer itself has no pathognomonic sign. It is practically without symptomatology. The symptoms ordinarily spoken of as pertaining to it are those induced on other tissues by its presence. But while insidious in its onset, advancing at first with "velvet tread," still in the majority of cases certain definite signs are evident early; "danger signals" as Childe calls them, are thrown out. And it is of these signs that the public must be taught to be observant, and of which the physician must appreciate the full significance.

In cancer of the breast the danger signal is a small lump. 80% of the tumors of the breast are or will eventually become malignant, (Beckman), and in women over 35 to 40 years of age they are malignant in 90% of the cases (Childe). The lump may be in any part of the breast, but usually it is in the outer half and most commonly in the upper and outer quadrant. Retraction of the nipple so generally considered a pathognomonic sign is not constant, occurring in but 51% of the cases (Rodman). In about 10% of the cases cancer of the breast cannot be recognized clinically (Rodman). Our duty then is plain and imperative. To temporize, to wait to see whether more definite signs develop is probably robbing the patient of her chance for cure.

In cancer of the uterus the danger signal is slight, irregular bleedings at irregular times. Quoting from the report of the Committee on Cancer of the Uterus of the American Medical Association, 1906, "Bleeding or blood stained discharge is usually but not always present. It may be: (1) Slight, only a show, appearing at irregular intervals, as on exertion, after sexual intercourse, using a douche or straining at stool; or it may be slight, but constant, the patient noticing that her clothes are slightly stained on taking them off at night. (2) In other cases the bleeding may be more profuse, simulating a prolonged or irregular menstruation or a return of the menses after the menopause. (3) In still other cases severe hemorrhage may occur, appearing either as the result of some unusual exertion, or during menstruation, or the cause may not be apparent.

"In a small percentage of the cases bleeding may be absent, but usually some other sign, such as an unusual leucorrhoeal discharge, calls attention to the growth. In a small percentage of cases all symptoms referable to the growth may be absent for a long time. Pain caused by the growth usually occurs later in the course of the disease and must be differentiated sharply from pain arising from pelvic trouble independent of the cancer, such as inflammatory conditions of the tubes, ovaries, etc."

The profession itself has been largely at fault for the prevailing popular idea that irregular bleedings at the menopause and after are normal and of no particular significance. No woman with the danger signal present should be turned away without a most thorough examination. Cullen says "general practitioners and surgeons have absolutely no excuse for failing to diagnose cancer of the uterus within one week after the first time the patient comes under observation. If the clinical signs are not definite a small wedge from a suspected cervix or curettings from a suspected endometrium, submitted to microscopic examination, will establish the diagnosis."

In câncer of the lip, mouth or tongue the danger signal is a sore which does not promptly heal, a wart or leukoplakia. No one, even the most unobservant, can fail to be aware of the warning sign at these sites. Many times each day it makes its presence known. Such a sore after 40 years of age, is almost surely cancerous, the wart is either malignant at the start or soon becomes so, and frequently the leukoplakia undergoes malignant change.

In cancer of the skin the danger signal in persons over 35 or 40 years of age is a sore at any site which does not heal or a wart or a mole which takes on any change. W. W. Keen and others have insisted on the advisability of removal of warts and moles in adults before they have a chance to take on malignant change and this would certainly be the part of wisdom, particularly, where they are subject to any irritation.

In cancer of the stomach danger signals are not so evident. However, uneasiness in the epigastrium after eating and loss of appetite, and most certainly persistent pain and any loss of flesh and strength should lead a person past middle life to a physician at once. Rodman says "diagnosis is difficult and uncertain and this is the strongest plea that can be made for an early exploratory incision in all gastric cases of doubtful nature, failing to yield to medical treatment in a reasonable time." Three or four weeks seem a fair period for such a trial of non-surgical treatment.

As to the large intestine, the danger signals are persistent colicky pain in the abdomen, obstinate constipation or alternating constipation and diarrhoea, and bloody stools. These signs merit the most careful attention by the practitioner, a thorough X-Ray investigation and surgical consultation. Most unfortunate is the common tendency of patients to ascribe any rectal symptoms to "piles" and most reprehensible and culpable is it for the physician to fail to make a local examination when piles are complained of.

With our present limited knowledge of cancer itself we are somewhat at a loss to know what measures to take directed toward its prevention. However, in this we are not altogether without some grounds for procedure. Enough evidence as to its infectivity has been adduced, to render it advisable that direct contact with cancerous lesions be avoided as far as possible, that discharges from external cancers should be taken up by suitable dressings and these burned, that where the lesion is in the mouth paper sputum cups should be used and disposed of by burning, and that rooms occupied by cancer patients should on their death be fumigated. Chronic irritation at certain sites does we know predispose to cancer. Therefore avoidance of such irritation is the indication. For example, ill fitting corsets, producing repeated small traumatisms of the breast should not be worn; old broken and decayed teeth should be taken care of and the mouth kept in a hygienic condition; smokers in later life should beware of inducing chronic soreness of the mouth or tongue; picking at moles or warts should be avoided. Finally, in the matter of prevention, such treatments or operations should be undertaken as will rid patients particularly those past middle life, of all tumors of the breast, of leucorrhoea (attacking the underlying endometritis, cervical erosion or other cause), of ulcers of the stomach, of intractable sores anywhere, and of moles and warts, particularly, those which are exposed to irritation.

When malignant change has occurred, when cancer is present, there is but one treatment which offers a fair chance of cure. That treatment is early and complete surgical removal. There is but one exception to this statement and that is in the case of rodent ulcers where in the hands of experts the X-Ray has proved its efficiency. It is not enough that simply the evident lesion be excised. Caustics for ages have accomplished this much but curative they have not proved themselves. The removal must be complete and by complete we mean by block dissection with removal of adjacent glands after the manner of a Halsted breast operation or a Crile neck dissection. Having some curative power and certainly helpful in palliation, may be mentioned in addition, the X-Ray, radium and fulguration. These however, are methods to be employed where for some reason radical removal is not possible.

In conclusion, as Childe says "Early diagnosis and treatment can be obtained in no other way than by education, both within the profession and outside of it; by placing in the hands of the intelligent and educated classes the knowledge that will enable them to avoid delay themselves, and by instructing those who come in contact with the uneducated classes, so that they may be in a position to help them in their hour of need." It is for each of us to disseminate judiciously such knowledge of cancer that the public may come to an appreciation of the following facts, as stated by Childe and others:

(1) That cancer is at first local.

(2) That it soon spreads to adjacent and distant parts.

(3) That in a person over 35 or 40 years of age " a lump in the breast, a sore in the mouth, an intractable sore on the skin, an irregular vaginal hemorrhage, severe and intractable digestive trouble, is more probably cancer than anything else.''

(4) That cancer is curable by surgery.

References:

''The Control of a Scourge'' by Geo. P. Childe, published by Dutton & Co., of N. Y.

John A. McGlinn, Monthly Cyclopedia and Medical Bulletin, April, 1909.

E. H. Beckman, Journal of Minnesota State Medical Association and the Northwestern Lancet, December 15, 1909.

S. P. Beebe, N. Y. Medical Journal, May 14, 1915.

W. S. Halsted, Annals of Surgery, July, 1907.

J. C. Bloodgood, American Journal Med. Sciences, vol. CXLVII, 76, 1914.

J. M. Wainwright, Pennsylvania Medical Journal, August, 1909.

W. L. Rodman, Pennsylvania Medical Journal, November, 1909.

F. S. Cullen, Pennsylvania Medical Journal, November, 1909

W. W. Keen, Transactions of the section on surgery and anatomy of the American Medical Association, 1904.

186 Linwood Avenue.

---

**Anuria in Typhoid.** Stephen Harberger (note the appropriateness of the name and subject) of Catlett, Va., Charlotte Med. Jour., May, 1915. References in literature are meagre, alluding merely to rarity and severity of the condition. In 1908, ten of eleven persons using a well in the outskirts developed typhoid. Three of four white and three of six colored persons developed anuria. Two of the white cases died, one a woman aged 59, the other a girl of 16. The three colored cases, in boys 4-7, recovered. The 16-year old white girl had, at one time, a temperature of 107. Colon flushings, hot packs and steam baths were used. This epidemic was traced to a convalescent visitor from Washington.

---

**Caramel in Diabetes.** Grafe, Münch. Med. Woch., 1914, claims that this is well tolerated, lessening acidosis without tending to increase glycosuria 100-200 grams may be given daily. It may be prepared by heating glucose in an aluminum dish to 140-150 C. (Note: This does not require elaborate apparatus. A tin oven from an oil or gas stove may be used, with a laboratory thermometer inserted through a hole in cork fitted in the top. Heat by Bunsen burner not placed directly under the dish and place the latter on thick asbestos to insure against too high local temperature).

